# Systematic Review and Geospatial Modeling of Molecular Markers of Resistance to Artemisinins and Sulfadoxine–Pyrimethamine in *Plasmodium falciparum* in India

**DOI:** 10.4269/ajtmh.23-0631

**Published:** 2024-04-02

**Authors:** Minu Nain, Mehul Dhorda, Jennifer A. Flegg, Apoorv Gupta, Lucinda E. Harrison, Sauman Singh-Phulgenda, Sabina D. Otienoburu, Eli Harriss, Praveen K. Bharti, Beauty Behera, Manju Rahi, Philippe J. Guerin, Amit Sharma

**Affiliations:** ^1^ ICMR-National Institute of Malaria Research, New Delhi, India;; ^2^ WorldWide Antimalarial Resistance Network, Oxford, United Kingdom;; ^3^ Infectious Diseases Data Observatory, Oxford, United Kingdom;; ^4^ Mahidol Oxford Tropical Medicine Research Unit, Faculty of Tropical Medicine, Mahidol University, Bangkok, Thailand;; ^5^ Centre for Tropical Medicine and Global Health, Nuffield Department of Medicine, University of Oxford, Oxford, United Kingdom;; ^6^ School of Mathematics and Statistics, University of Melbourne, Parkville, Victoria, Australia;; ^7^ College of STEM, Johnson C. Smith University, Charlotte, North Carolina;; ^8^ The Knowledge Centre, Bodleian Health Care Libraries, University of Oxford, Oxford, United Kingdom;; ^9^ Indian Council of Medical Research, New Delhi, India;; ^10^ Academy of Scientific and Innovative Research, Ghaziabad, Uttar Pradesh;; ^11^ Molecular Medicine, International Centre for Genetic Engineering and Biotechnology, New Delhi, India

## Abstract

Surveillance for genetic markers of resistance can provide valuable information on the likely efficacy of antimalarials but needs to be targeted to ensure optimal use of resources. We conducted a systematic search and review of publications in seven databases to compile resistance marker data from studies in India. The sample collection from the studies identified from this search was conducted between 1994 and 2020, and these studies were published between 1994 and 2022. In all, *Plasmodium falciparum* Kelch13 (PfK13), *P. falciparum* dihydropteroate synthase, and *P. falciparum* dihydrofolate reductase (PfDHPS) genotype data from 2,953, 4,148, and 4,222 blood samples from patients with laboratory-confirmed malaria, respectively, were extracted from these publications and uploaded onto the WorldWide Antimalarial Resistance Network molecular surveyors. These data were fed into hierarchical geostatistical models to produce maps with a predicted prevalence of the PfK13 and PfDHPS markers, and of the associated uncertainty. Zones with a predicted PfDHPS 540E prevalence of >15% were identified in central, eastern, and northeastern India. The predicted prevalence of PfK13 mutants was nonzero at only a few locations, but were within or adjacent to the zones with >15% prevalence of PfDHPS 540E. There may be a greater probability of artesunate–sulfadoxine–pyrimethamine failures in these regions, but these predictions need confirmation. This work can be applied in India and elsewhere to help identify the treatments most likely to be effective for malaria elimination.

## INTRODUCTION

Despite century-long control and elimination efforts, malaria remains a major public health concern. The WHO has set an ambitious goal of malaria elimination in 35 countries and at least a 90% reduction in malaria cases by 2030.^1^^,^^2^ Within the WHO Southeast Asia region, India remains the leading contributor to the malaria burden, with 79% of cases and 83% of total malaria deaths.^3^ The proportion of *Plasmodium falciparum* and mixed infections in India has increased from 50% in 2010 to more than 60% in 2021.^3^ Under the auspices of the Global Technical Strategy, adopted by the World Health Assembly in May 2015, India launched its malaria elimination program in 2016 under the National Framework for Malaria Elimination in India 2016–2030.^1^^,^^4^ Under this framework, India aims to eliminate malaria, prevent reintroduction, and maintain malaria-free status across the country by 2030. Active surveillance of low-density/asymptomatic infections, malaria epidemiology (parasite and vector) studies, and tracking drug and insecticide resistance were identified as crucial to attaining this goal.

Artemisinin-based combination therapies (ACTs) are the first line of treatment of *P. falciparum* across the world.^3^ Sulfadoxine–pyrimethamine (SP) continues to be used for prophylaxis in certain regions and subpopulations and, along with artesunate, in an ACT to treat uncomplicated *P. falciparum* malaria in India, Iran, Afghanistan, Pakistan, Saudi Arabia, and Yemen.^5^^,^^6^ Artemisinin resistance related to mutations in *Plasmodium falciparum* Kelch13 (PfK13) has emerged and spread from multiple foci in recent years.^7^ There is increasing concern about ACT efficacy, because of the significant increase in the prevalence of PfK13 mutations in the Greater Mekong subregion (GMS), where only 65% of samples collected between 2015 and 2020 were Wild-type, with a high prevalence across the GMS of two mutations (R539T and C580Y) known to be strongly associated with the artemisinin resistance phenotype.^8^^,^^9^ For SP resistance, a combination of mutations in *Plasmodium falciparum* dihydropteroate synthase (PfDHPS) and *Plasmodium falciparum* dihydrofolate reductase (PfDHFR) in the form of quintuple and sextuple mutations leads to fully resistant and super-resistant phenotypes, respectively. The quintuple mutant includes three mutations in PfDHFR and two mutations in PfDHPS (N51I, C59R, S108N, A437G, and K540E). Artesunate-SP (AS-SP) failure rates of >10% and the high prevalence of resistant SP parasites, including quintuple-mutant haplotypes in northeastern India, led to a policy change for uncomplicated *P. falciparum* malaria treatment from AS-SP to artemether–lumefantrine (AL) in the states of Assam, Tripura, Meghalaya, Mizoram, and Manipur, and the Arunachal Pradesh region in 2014.^5^^,^^10^^–^^14^

Since then, SP resistance, including single, double, triple, and, quadruple mutations in PfDHFR and PfDHPS, has been detected in other parts of the country as well.^15^^–^^23^ The emergence and spread of artemisinin resistance in India could be expected to lead to the selection of partner drug-resistant parasites. This might lead to complete therapeutic failure of not only AS-SP, but also of AL.^13^^,^^14^ Artemisinin resistance is hence one of the biggest threats to the malaria elimination program in India and elsewhere.

Large-scale active surveillance is crucial to obtain precise information on the emergence and spread of antimalaria-resistant mutations. This need has to be optimized against available resources such that information is collected in the most efficient manner and is made available to relevant stakeholders. The representativeness of sites where surveillance is to be conducted is a challenging methodological question. Geospatial modeling has been proposed as a feasible statistical method to address site selection issues.^24^^,^^25^ It can take into account the already known information on the resistance markers to get a precise map of geospatial trends in the prevalence of drug resistance genes.^26^ The information generated from these geospatial models can be used to design optimal strategies for future molecular surveillance of drug resistance.

In our systematic review, we aim to compile the available information from published and unpublished literature on the prevalence of resistance markers across the malaria-endemic states of India. We used a mathematical model to predict the prevalence of artemisinin- and SP-resistant parasites even in locations where no data have been collected, and to identify the regions with the most uncertainty or imprecision in the predicted prevalence. This model can eventually be applied to identify locations where more genotyping data need to be collected, thus helping to target future surveillance studies in a more systematic way.

## MATERIALS AND METHODS

### Study design.

Our study has two major components: a systematic review of the prevalence of drug resistance markers and a spatiotemporal analysis of available data. The data presented and analyzed here have been extracted from published and unpublished studies on the molecular prevalence of *P. falciparum* markers of resistance to SP (PfDHPS, PfDHFR) and to artemisinin (PfK13) across India up to March 2022. The extracted data were also used to update the previously established databases of PfDHPS*/*PfDHFR and PfK13 resistance markers on the WorldWide Antimalarial Resistance Network (WWARN) molecular surveyors,^27^^,^^28^ and to generate a map of the distribution of PfDHPS/PfDHFR and PfK13 mutations across India.

### Criteria for study selection.

The studies were selected for the systematic review and spatiotemporal analysis using predefined criteria, as summarized in Supplemental Table 1. Also, the already available Indian studies from the WWARN database were included.

### Search strategy.

Studies on PfK13 and PfDHPS/PfDHFR were searched on the following databases: Ovid Embase, Ovid Medline, Scopus, clinicaltrials.gov, Web of Science Core Collection, WHO–International Clinical Trials Registry Platform, and the Cochrane Central Register of Controlled Trials. The search was run by an experienced librarian at the Bodleian Health Care Libraries, at the University of Oxford, to collect all studies based on the designed search strategy registered or published from January 2014 to March 2022. The search strategies are available in full (Supplemental Appendixes 1 and 2). Studies were limited to those published in English, French, Spanish, Italian, or Hindi. All references were exported to Endnote v. X9 (Thomson Reuters, New York, NY), and duplicates were removed manually.

### Study screening.

The studies identified from databases were screened using Covidence systematic review software (Veritas Health Innovations, Melbourne, Australia), an online screening and data extraction tool. Three reviewers screened the studies identified in the search against the prespecified eligibility criteria in a blinded fashion. The primary reviewer screened all the studies identified using the described search strategy per the predefined criteria. All studies retained by the primary reviewer were distributed in a random, blinded manner between two additional reviewers. Disagreements among the reviewers were discussed until a consensus was reached. All studies conducted in India and not already included in the WWARN database were selected for full-text screening. Data were extracted only from those studies that reported complete information on the location and period of sample collection, basic demographic data, and the prevalence of the markers of interest. Studies excluded during the full-text screening are summarized in Supplemental Table 2.

### Data extraction.

Data on the following aspects of the study were recorded: study title, author details, study year (precise or estimated), publication year, geographic location (precise or estimated), age group, gender, and number of successfully sequenced samples for each study site; the number of samples with Wild-type parasites and with WHO-validated single nucleotide polymorphisms (SNPs) associated with SP resistance (PfDHPS/PfDHFR) or artemisinin resistance (PfK13) were also recorded. Data submitted by the authors of an unpublished study conducted in Odisha, India, were also extracted. All extracted data were uploaded into the WWARN database for display on the SP and the artemisinin resistance surveyors.^27^^,^^28^ Validity and risk of bias among studies and in overall data extracted were mitigated by adhering to the study methods (eligibility, data extraction, and analysis).

### Modeling.

The updated data set was used for spatiotemporal modeling.^27^^,^^28^ The strains with the PfDHPS 540E mutation rarely occur independently, and the mutation occurs mainly in combination with other PfDHPS and PfDHFR mutations in the form of quintuple and sextuple mutations. Hence, PfDHPS 540E is generally used as a proxy for quintuple and sextuple PfDHFR/PfDHPS mutants associated with clinical SP resistance.^29^^,^^30^ The prevalence of the PfDHPS 540E mutation and the overall prevalence of any PfK13 mutation associated with delayed parasite clearance were calculated based on data downloaded from the WWARN surveyors in October and November 2022, respectively.^31^^,^^32^ Each data point was combined within a hierarchical geostatistical model to produce predictive maps of the prevalence of the PfDHPS 540E and PfK13 markers across all malaria-endemic regions of India.

The statistical methodology followed two stages to allow for spatiotemporal prediction of the molecular marker prevalence, as documented previously.^24^^–^^26^^,^^33^ First, based on the observed data, the posterior distribution of model parameters was estimated using a Bayesian inference framework. Second, given the model parameters from the first stage, marker prevalence was predicted on a 5- × 5-km grid over India. For each location, the distribution of prevalence was drawn from the posterior predictive distribution and summarized using the median statistic to create a point estimate surface. The SD surface of the posterior predictive distribution was presented alongside the median maps as a descriptor of the associated uncertainty in the predictions at each location.

## RESULTS

### Number of studies.

**Figure 1.**
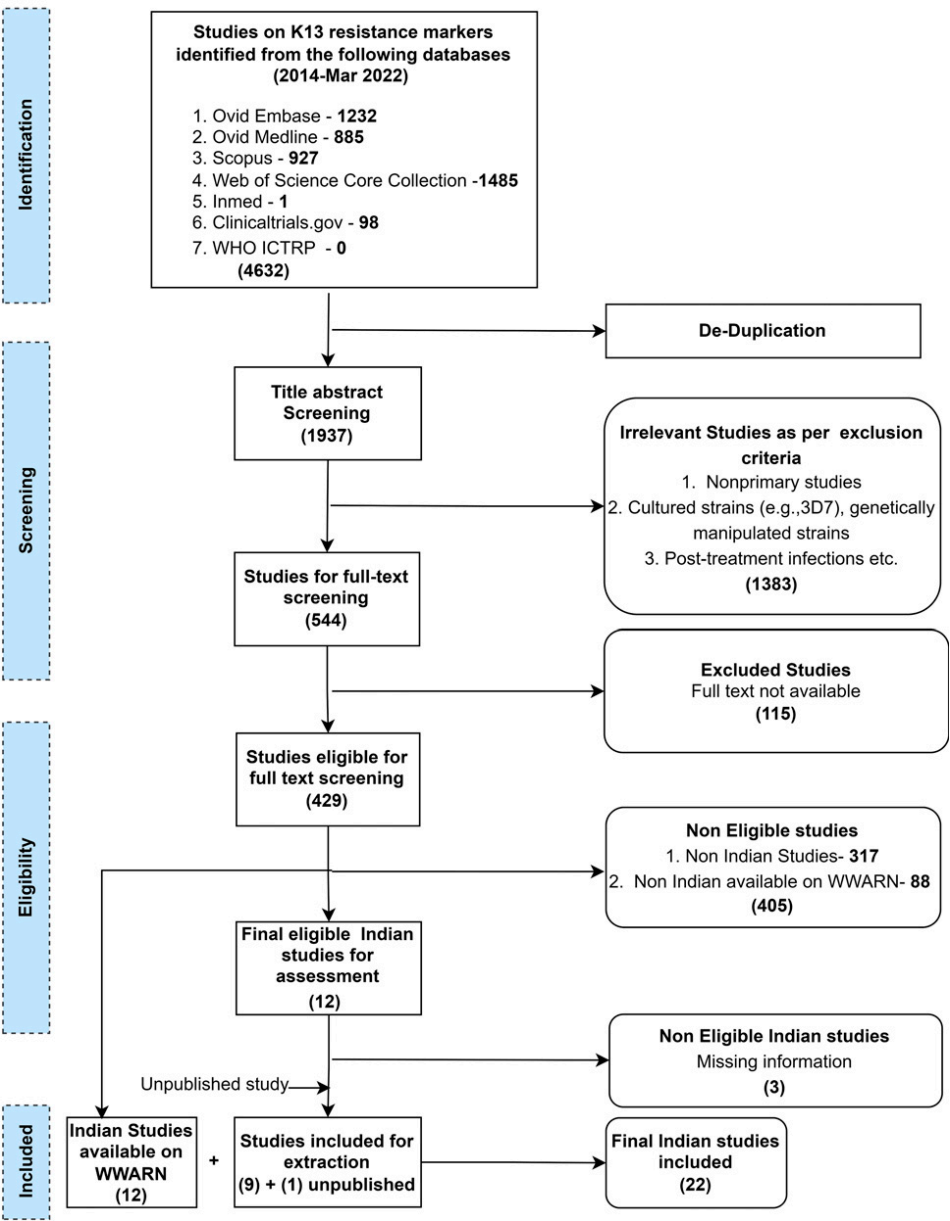
Workflow for study selection of *Plasmodium falciparum* Kelch13 (K13) resistance marker studies from India. ICTRP = International Clinical Trials Registry Platform; WWARN = WorldWide Antimalarial Resistance Network.

**Figure 2.**
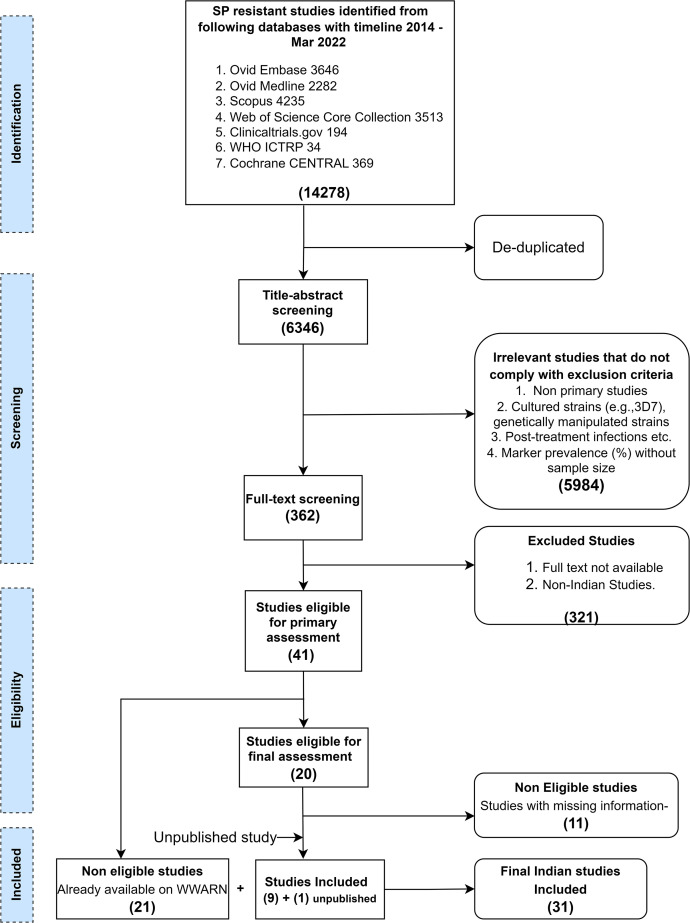
Workflow for study selection *Plasmodium falciparum* dihydropteroate synthase/*P. falciparum* dihydrofolate reductase resistance marker studies from India. ICTRP = International Clinical Trials Registry Platform; SP = sulfadoxine–pyrimethamine; WWARN = WorldWide Antimalarial Resistance Network.

A total of 1,927 and 14,278 publications were retrieved from the databases for the artemisinin (PfK13) and SP (PfDHPS/PfDHFR) resistance markers, respectively. For both markers, data from one unpublished study were also included. In total, data were extracted from 22 PfK13- and 31 PfDHPS/PfDHFR-relevant Indian studies inclusive of newly retrieved as well as already available studies on the WWARN database from 2014 until March 2022. Details of the study selection process for PfK13 and PfDHPS/PfDHFR are presented in Figures 1 and 2, respectively. The individual study results are summarized and available on the WWARN artemisinin and SP surveyors (WWARN.org).

### Molecular studies as part of a trial versus pure surveillance.

**Figure 3.**
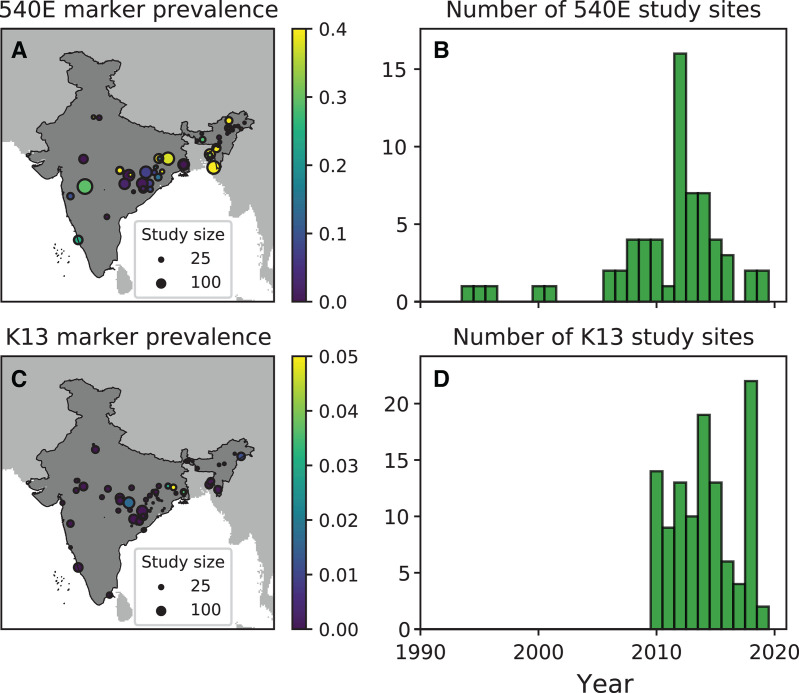
(**A**) Map showing the spatial location of the studies included in the modeling of *Plasmodium falciparum* dihydropteroate synthase (PfDHPS) 540E prevalence. The size of the marker is proportional to the number of patients in the study; the color of the marker denotes the observed marker prevalence. (**B**) The number of PfDHPS 540E study sites each year. (**C**) Map showing the spatial location of the studies included in the modeling of *P. falciparum* Kelch13 (K13) prevalence. The size of the marker is proportional to the number of patients in the study; the color of the marker denotes the observed marker prevalence. (**D**) The number of *P. falciparum* K13 study sites each year.

A total of 3,040 samples from 22 studies were sequenced for PfK13 across India either as a part of a genetic surveillance study or a therapeutic efficacy trial (Figures 1 and 3C, Supplemental Table 3). Of the included 22 studies, 9 were part of ACT therapeutic efficacy trials; 11 were genetic surveillance studies, of which 1 was on imported cases in Australia; 1 was a case study; and 1 was a pharmacokinetics trial for artemisinin.

For PfDHPS/PfDHFR markers, 4,148 PfDHFR and 4,222 PfDHPS samples from 31 studies were sequenced. Of these 31 studies, 23 were genetic surveillance studies and the remaining 8 were therapeutic efficacy studies (Figures 2 and 3A, Supplemental Table 4). Sanger sequencing is the most commonly used genotyping technique in these studies (Supplemental Tables 3 and 4). The median time lag between the start of sample collection and study publication for PfK13 and PfDHPS/PfDHFR was 4 years.

### Geographic distribution.

**Figure 4.**
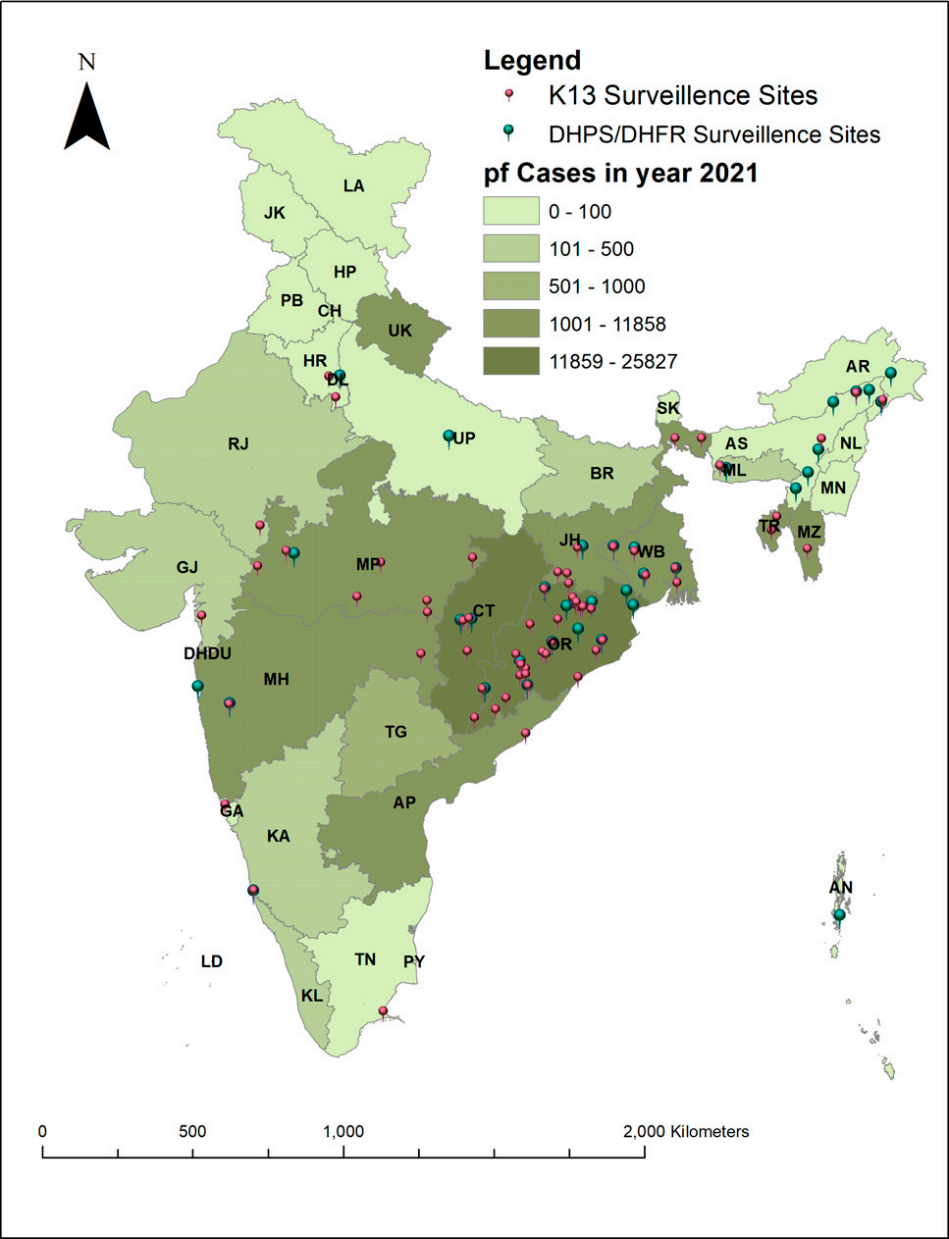
Correlation between malaria endemicity in 2021 and number of *Plasmodium falciparum* Kelch13 (K13) and *P. falciparum* dihydropteroate synthase (DHPS)/*P. falciparum* dihydrofolate reductase (DHFR) prevalence studies conducted in India until March 2022. AN = Andaman and Nicobar Islands; AP = Andhra Pradesh; AR = Arunachal Pradesh; AS = Assam; BR = Bihar; CH = Chandigarh; CT = Chhattisgarh; DN = Dadra and Nagar Haveli; DD = Daman and Diu; DL = Delhi; GA = Goa; GJ = Gujarat; HR = Haryana; HP = Himachal Pradesh; JK = Jammu and Kashmir; JH = Jharkhand; KA = Karnataka; KL = Kerala; LD = Lashadweep; MP = Madhya Pradesh; MH = Maharashtra; MN = Manipur; ML = Meghalaya; MZ = Mizoram; NL = Nagaland; OR = Orissa; PY = Puducherry; PB = Punjab; RJ = Rajasthan; SK = Sikkim; TN = Tamil Nadu; TG = Telangana; TR = Tripura; UP = Uttar Pradesh; UK = Uttarakhand; WB = West Bengal. pf = *Plasmodium falciparum.*

Malaria is endemic in large parts of India, but with varying levels of prevalence or incidence. However, both the number of studies and their geographic coverage were small or limited. Per the latest epidemiological data of 2020 and 2021, malaria is reported across India, with the maximum number of falciparum cases (>1,000 cases/year) from Odisha, Chhattisgarh, Madhya Pradesh, Maharashtra, West Bengal, Jharkhand, Uttarakhand, Andhra Pradesh, Tripura, and Mizoram (Figure 4, Supplemental Table 5). Apart from these states, the other states with reported cases in the range of 50–1,000 cases/year include Assam, Bihar, Gujarat, Karnataka, Kerala, Meghalaya, Rajasthan, Telangana, and Tamil Nadu. The states with low endemicity reporting <50 cases/year include Andaman and Nicobar, Delhi, and Goa (Figure 4, Supplemental Table 5).

For PfK13, the maximum number of studies (*n* = 4) conducted in West Bengal, followed by three studies in Chhattisgarh and Odisha, and two studies each from Tripura, Delhi, Maharashtra, and Madhya Pradesh. There were only three studies reported from northeastern India (two from Tripura and one from Arunachal Pradesh) and one study each from Goa, Karnataka, and Tamil Nadu. There were no published surveillance studies from Andhra Pradesh and Uttarakhand (Figures 3C and 4). The earliest studies were conducted in 2015 in the states of Jharkhand, Assam, Meghalaya, Tripura, and Mizoram, with the most recent study being from 2019 in Odisha.

The studies on the prevalence of SP resistance patterns show a similarly skewed geographic distribution. The maximum number studies (*n* = 7) were reported from Odisha, followed by three studies from West Bengal and two studies each from Maharashtra, Chhattisgarh, Assam, and Arunachal Pradesh (Figures 3A and 4). Only one study was reported from each of the other endemic states: Delhi, Madhya Pradesh, Karnataka, Jharkhand, Tripura, Uttar Pradesh, Andhra Pradesh, and the Andaman and Nicobar islands (Figures 3A and 4). The earliest studies reporting data on PfDHFR/PfDHPS were conducted in 2011 in Andhra Pradesh, whereas the most recent study mutations were conducted in 2019 in Odisha. The timeline for the last surveillance study conducted from the states with maximum *P. falciparum* cases, reported in 2020–2021 is presented in Table 1.

**Table 1:** The timeline (year) of the last surveillance study for the prevalence of *Plasmodium falciparum* Kelch13 and *P. falciparum* dihydropteroate synthase/*P. falciparum* dihydrofolate reductase markers conducted across the high-burden malaria states of India

State	*pfdhps*/*pfdhfr* Marker Surveillance Year	*pfk13* Marker Surveillance Year
Odisha	2019	2019
Chhattisgarh	2017	2018
Madhya Pradesh	2016	2017
Maharashtra	2012	2017
West Bengal	2016	2016
Jharkhand	2016	2015
Tripura	2015	2015
Mizoram	2012	2015
Andhra Pradesh	2011	–
Uttarakhand	–	–

*pfdhfr* = *Plasmodium falciparum* dihydrofolate reductase; *pfdhps* = *P. falciparum* dihydropteroate synthase; *pkf13* = *P. falciparum* Kelch13.

### Limited surveillance studies.

The maximum number of surveillance studies were conducted in the three states with the greatest malaria endemicity: Chhattisgarh, West Bengal, and Odisha. In states such as Maharashtra, the number of *P. falciparum* cases increased from 2,697 in 2018 to 11,858 in 2021. Despite this increase, there are only two PfK13 and two PfDHPS/PfDHFR surveillance studies published since 2014 from that state (Figures 3C and 4).^12^^,^^34^^,^^35^ Collectively, only 145 and 200 samples were genotyped successfully for PfK13 and PfDHPS/PfDHFR, respectively, from the available studies. Similarly, a very limited number of samples has been tested for PfDHPS/PfDHFR mutations from the states of Jharkhand (84 samples) and Uttar Pradesh (31 samples) between 2006 and 2015.^36^ No PfDHPS/PfDHFR studies have been reported from the state of Andhra Pradesh since 2012, and there are no studies from Bihar, Kerala, Gujarat, Tamil Nadu, Rajasthan, Telangana, and Uttarakhand (Figures 3A and 4). Similarly, hardly any PfK13 studies have been reported from these states, despite a significant number of *P. falciparum* cases reported from them per 2021 malaria endemicity data in India (Figure 4).

### Uncertainty in PfK13 and PfDHPS/PfDHFR predicted prevalence.

**Figure 5.**
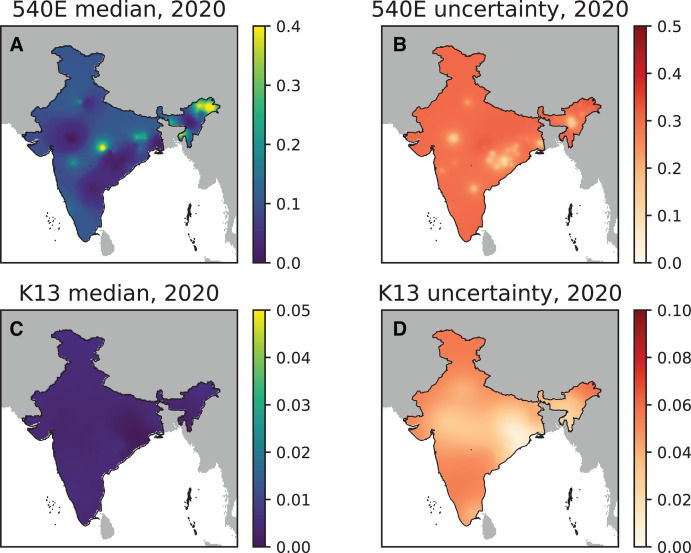
The posterior predictive median prevalence of *Plasmodium falciparum* dihydropteroate synthase (PfDHPS) 540E in India in 2020 (**A**) and the associated SD for posterior predictions in 2020 (**B**). The posterior predictive median prevalence of *P. falciparum* Kelch13 (K13) in India in 2020 (**C**) and the associated SD for posterior predictions in 2020 (**D**).

All the studies published up to March 2022 with samples collected from 2010 onward were included in the geospatial model for PfK13 (Figure 3D). Most of the studies are concentrated in the states of West Bengal, Chhattisgarh, Odisha, Jharkhand, Madhya Pradesh, Assam, Meghalaya, and Mizoram, with the greatest prevalence of PfK13 mutants being in West Bengal, Madhya Pradesh, and Arunachal Pradesh (Figure 3C). The calculated median and predicted uncertainty on the prevalence of PfK13 resistance markers are high in Arunachal Pradesh and Assam (Figure 5C and D). The studies of PfK13 markers from other parts of the country were not sufficient to estimate the PfK13 prevalence and thus could not identify the regions with high uncertainty (Figure 5C and D).

**Figure 6.**
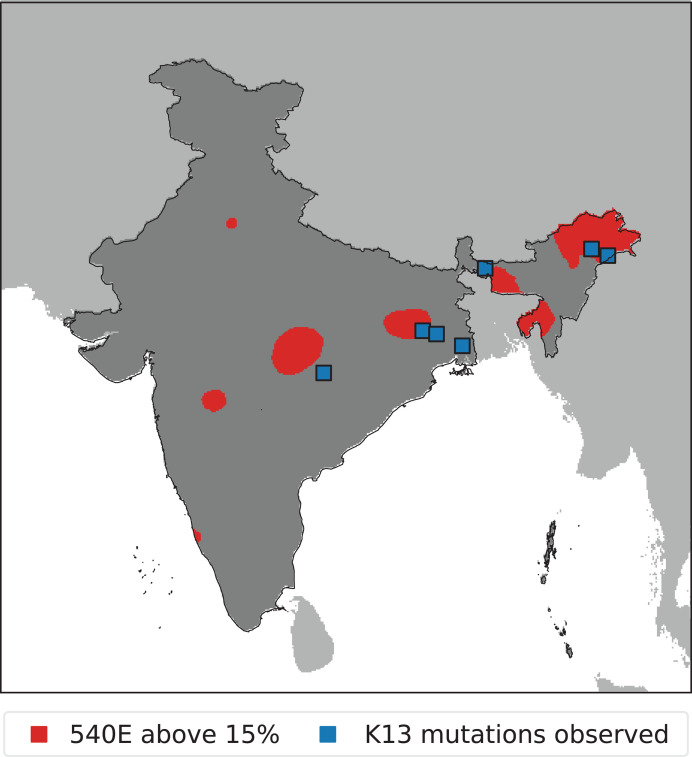
The predicted areas with *Plasmodium falciparum* dihydropteroate synthase 540E marker prevalence exceeding 15% (red shading) based on median predictions in 2020, and the locations of *P. falciparum* Kelch13 (K13) studies that had a nonzero PfK13 prevalence observed in the data collected.

For SP markers, all studies published until March 2022 with a sample collection timeline from 1994 onward were included (Figure 3B). More studies were conducted for PfDHPS/PfDHFR markers compared with PfK13. However, like PfK13, most of the PfDHPS/PfDHFR studies are from the states of West Bengal, Jharkhand, Chhattisgarh, Odisha, Madhya Pradesh, Assam, Arunachal Pradesh, Mizoram, and Tripura, with a maximum PfDHPS 540E prevalence of >5% in all the northeastern states and emerging resistance markers in other states, including West Bengal, Maharashtra, Madhya Pradesh, Delhi, Odisha, and Jharkhand (Figures 5A and 6). The states with high uncertainty in PfDHPS/PfDHFR prevalence data include Assam, Arunachal Pradesh, Meghalaya, Tripura, Mizoram, West Bengal, Maharashtra, Madhya Pradesh, Gujarat, Andhra Pradesh, and Uttarakhand (Figure 5B).

### The population at risk for resistant parasites.

Although the reports of validated PfK13 mutations from India are still limited, there are several reports of nonsynonymous SNPs, some of which are candidate resistance markers as well. Most significant of these SNPs (including the G625R, R539T, and F446I polymorphisms), associated with in vivo and in vitro artemisinin resistance phenotypes, were reported from West Bengal and Arunachal Pradesh,^37^^–^^40^ whereas the nonsynonymous mutation A578S has been reported from Mizoram in northeastern India.^39^ Apart from these SNPs associated with delayed parasite clearance, there are reports of multiple nonsynonymous mutations, not associated with delayed parasite clearance to date, from regions of eastern and central India, including Madhya Pradesh, Odisha, Jharkhand, Chhattisgarh, West Bengal, Tripura, Assam, Arunachal Pradesh, and Mizoram (Figures 5C and 6).^15^^,^^41^^–^^44^ The combination of malaria endemicity (Figure 4), proximity to the GMS, and population movements together with the presence of the nonsynonymous SNPs put the population of these states at risk of encountering ACT failure in the future. For PfDHFR, 59R, 108N, and 437G are the most common SNPs present either alone (single) or in combination (double or triple) as multiple haplotypes. Similarly, for PfDHPS, 437G and 540E are the most common SNPs present across India. However, a limited number of therapeutic efficacy studies from the rest of India show that AS-SP remains effective despite the presence of SP resistance,^43^^,^^45^^,^^46^ except for a report mentioning AS-SP failure in ∼4% of cases from central India in 2015 to 2017.^15^ Hence, the prevalence of PfK13 resistance markers in the regions of Assam, Arunachal Pradesh, West Bengal, Jharkhand, and Chhattisgarh with a high prevalence of PfDHPS/PfDHFR markers (>15%) in the proximal regions (Figure 6) put these states and other endemic neighboring states at the risk of the emergence and spread of multidrug resistance in future.

## DISCUSSION

In our systematic review, we analyzed the prevalence of artemisinin and SP resistance markers in India based on available literature. We found there are limited surveillance and even fewer published therapeutic studies from India when compared with other neighboring Southeast Asian countries, with almost double the number of studies conducted compared with India, despite being one of the countries with a major malaria burden. The number of studies in correlation with malaria endemicity shows that the study distribution is not uniform across the endemic states. Most studies are concentrated in limited regions with the greatest malaria burden. There is a need for widescale surveillance studies covering the vast geographic and ecological diversity of the country, including not only the highly endemic regions, but also those with moderate or low endemicity. Regions with low endemicity may be particularly important for resistance surveillance. As a result of low immunity in the population in such regions, a greater proportion of infections are likely to cause a clinical episode, increasing the likelihood of treatment and hence selective pressure on the parasites.

A geospatial mathematical model based on the data extracted on PfK13 and PfDHPS/PfDHFR markers was used in our study to estimate the distribution of drug-resistant parasites outside of locations where studies were conducted. The model outputs include the uncertainty of the estimated prevalence of drug-resistant parasites that can be used to identify spots where data are scarce, and hence where surveillance is most needed. Contrary to expectation, some of the regions with the greatest uncertainty in the predicted PfK13 and/or PfDHFR/PfDHPS prevalence of mutations, notably in the northeastern states, overlap with those where a relatively high number of studies has been conducted. This is likely a result of contradictory information available from those regions resulting from multiple reasons, as in some cases from studies conducted many years apart or Sanger missed mutations/rare mutations/low-frequency mutations. In most other regions, however, the uncertainty is a direct result of the scarcity of data from those regions. In either case, the principle of targeting surveillance studies in zones with the greatest uncertainty in the predicted prevalence remains valid—in the first case to confirm (or refute) the presence of resistant parasites and, in the second, simply to collect more molecular surveillance data.

Multiple SP-resistant haplotypes have been documented in the past from the northeastern states including quintuple, quadruple, and triple mutations from the states of Arunachal Pradesh, Tripura, and Assam.^13^^,^^38^^,^^39^^,^^47^ Since then, SP-resistant parasites with multiple PfDHPS/PfDHFR haplotypes have been identified across India, showing the spread of resistant parasites from northeastern India. Based on these data, the mathematical model identified zones with a high predicted prevalence of PfDHFR/PfDHPS mutations in regions of central, eastern, and northeastern India (>15%) (Figure 6). The measured and predicted prevalence of PfK13 mutants appears to have remained very low outside of the northeastern states. The locations where PfK13 mutant prevalence values were greater than zero are either within or adjacent to the zones with high-prevalence SP-resistant parasites. Therapeutic efficacies with AS-SP of <90% (the threshold for drug policy change) have not been reported to date outside northeastern India. In this context with a low prevalence of PfK13 mutations and high transmission, the combined effect of efficacious artemisinin and partial immunity may be sufficient to clear infections even with parasites with reduced susceptibility to SP.^48^ However, the high level of SP resistance likely translates into greater selective pressure favoring artemisinin resistance and increasing the risk of its emergence, or its spread if it has already emerged. If the widespread prevalence of parasites resistant to artemisinins and/or SP is confirmed through future studies, it could provide a strong impetus for a review of existing national drug policies, possibly leading to the nationwide deployment of other ACTs such as AL, which is the first-line treatment only in the northeastern states.^14^

The most recent data identified from the systematic review are from southern Odisha, where samples were collected in 2019. No published data collected after 2015 are available from the northeastern states, where genotypic and phenotypic evidence of artemisinin resistance has been detected. There is no information available on the presence or absence of PfK13, PfDHFR, and PfDHPS mutant parasites from Uttarakhand. These examples serve to highlight the very sparse coverage of the available data on which the geospatial model is constructed. It necessarily limits the accuracy and precision of the model outputs, and hence they cannot be considered as confirmation of drug resistance and/or treatment failures in regions with a predicted high prevalence of drug resistance markers. Furthermore, the uncertainty of the predicted values is affected by inconsistent data collected in the same areas. In some cases, such inconsistency could be a result of transient epiphenomena, but can also be a result of changes in the prevalence of the markers of interest in the same areas over time.^37^ The model as currently implemented does not take into account this temporal element. It can, however, be reasonably expected that these predictive outputs will improve with future iterations of the model that incorporate temporal data and, more importantly, if more data are collected systematically at regular intervals across the malaria-endemic regions of India.

## CONCLUSION

Our systematic review of the prevalence of mutations in PfK13, PfDHFR, and PfDHPS highlights the scarcity of the currently available data for these markers. Molecular surveillance can provide information rapidly on the likely efficacy of treatments. When incorporated into geospatial models, these data can improve the efficiency of surveillance efforts by helping to target such surveillance to zones with predicted resistance and/or from where information is sparse. Sustaining such systematic surveillance can provide useful evidence to support data-driven decisions on interventions most likely to be effective for malaria elimination from India and elsewhere.

## Supplemental Materials

10.4269/ajtmh.23-0631 Supplemental Materials

## Data Availability

The data that support the findings of this study are available for access via the WWARN (WWARN.org). The WWARN is registered with the Registry of Research Data Repositories (re3data.org).
