# Supplementary material for: Systematic Review and Geospatial Modeling of Molecular Markers of Resistance to Artemisinins and Sulfadoxine–Pyrimethamine in Plasmodium falciparum in India
Source: Am J Trop Med Hyg. 2024 Apr 2;110(5):910–20. doi: 10.4269/ajtmh.23-0631 (PMC11066343; doi:10.4269/ajtmh.23-0631)
Supplement: Supplemental Materials [file tpmd230631.SD1.pdf]

Molecular surveillance of Artemisinin resistance marker pfk13 prevalence in India

Search strategies run on 13/04/2022 by Eli Harriss, a librarian at the Bodleian Health Care Libraries, University of Oxford.

Search Results

Ovid Embase	1232
Ovid Medline	885
Scopus	927
Web of Science Core Collection	1489
IndMed	1
Clinicaltrials.gov	98
WHO ICTRP	0
Total	4632
Total after deduplication	1938

Search Strategies

Database: Embase 1974 to present
Search Strategy:

----

1 exp malaria/ (95255)
2 exp Plasmodium/ (64595)
3 (malaria or plasmodium).ti,ab,kw. (117440)
4 1 or 2 or 3 (137359)
5 (pfkelch* or pfk13* or k13 or kelch* or "artemisinin resistan*").ti,ab,kw. (4553)
6 4 and 5 (1520)
7 limit 6 to ((english or french or hindi or italian or spanish) and yr="2014 -Current") (1232)

Database: Medline (Ovid MEDLINE® Epub Ahead of Print, In-Process & Other Non-Indexed Citations, Ovid MEDLINE® Daily and Ovid MEDLINE®) 1946 to present
Search Strategy:

----

1 exp malaria/ (71391)
2 exp Plasmodium/ (50580)
3 (malaria or plasmodium).ti,ab,kw. (103372)
4 1 or 2 or 3 (112717)
5 (pfkelch* or pfk13* or k13 or kelch* or "artemisinin resistan*").ti,ab,kw. (3794)
6 4 and 5 (1070)
7 limit 6 to ((english or french or hindi or italian or spanish) and yr="2014 -Current") (885)

Scopus

(TITLE-ABS-KEY (malaria OR plasmodium) AND TITLE-ABS-KEY (pfkelch* OR pfk13* OR k13 OR kelch* OR "artemisinin resistan*")) AND (LIMIT-TO (PUBYEAR , 2022) OR LIMIT-TO (PUBYEAR , 2021) OR LIMIT-TO (PUBYEAR , 2020) OR LIMIT-TO (PUBYEAR , 2019) OR LIMIT-TO (PUBYEAR , 2018) OR

LIMIT-TO (PUBYEAR , 2017) OR LIMIT-TO (PUBYEAR , 2016) OR LIMIT-TO (PUBYEAR , 2015) OR LIMIT-TO (PUBYEAR , 2014)) AND (LIMIT-TO (LANGUAGE , "English") OR LIMIT-TO (LANGUAGE , "French"))

Web of Science Core Collection
malaria or plasmodium (Topic) AND pfkelch* or pfk13* or k13 or kelch* or "artemisinin resistan*" (Topic)
Refined by Publication Years: 2014 or 2022 or 2021 or 2020 or 2019 or 2018 or 2017 or 2016 or 2015

IndMed
https://www.india.gov.in/gsearch?s=pfkelch&op=Search

Pfkelch, pfk13, k13, kelch – no results
Artemisinin resistance – 1 result

Clinicaltrials.gov

Condition or disease: malaria
Other terms: pfkelch
All Studies

Condition or disease: malaria
Other terms: pfk13
All Studies

Condition or disease: malaria
Other terms: k13
All Studies

Condition or disease: malaria
Other terms: kelch
All Studies

Condition or disease: malaria
Other terms: artemisinin resistance
All Studies

WHO ICTRP https://trialsearch.who.int/
Trials registered from 2014/01/01 - 2022

Condition: Malaria
Intervention: kelch13 or pfk13 or k13 or kelch or artemisinin resistance
ALL studies

Molecular surveillance of Artemisinin resistance marker pfk13 prevalence in India

Search strategies run on 13/04/2022 by Eli Harriss, a librarian at the Bodleian Health Care Libraries, University of Oxford.

Search Results

Ovid Embase	1232
Ovid Medline	885
Scopus	927
Web of Science Core Collection	1489
IndMed	1
Clinicaltrials.gov	98
WHO ICTRP	0
Total	4632
Total after deduplication	1938

Search Strategies

Database: Embase 1974 to present
Search Strategy:
----
1 exp malaria/ (95255)
2 exp Plasmodium/ (64595)
3 (malaria or plasmodium).ti,ab,kw. (117440)
4 1 or 2 or 3 (137359)
5 (pfkelch* or pfk13* or k13 or kelch* or "artemisinin resistan*").ti,ab,kw. (4553)
6 4 and 5 (1520)
7 limit 6 to ((english or french or hindi or italian or spanish) and yr="2014 -Current") (1232)

Database: Medline (Ovid MEDLINE® Epub Ahead of Print, In-Process & Other Non-Indexed Citations, Ovid MEDLINE® Daily and Ovid MEDLINE®) 1946 to present
Search Strategy:
----
1 exp malaria/ (71391)
2 exp Plasmodium/ (50580)
3 (malaria or plasmodium).ti,ab,kw. (103372)
4 1 or 2 or 3 (112717)
5 (pfkelch* or pfk13* or k13 or kelch* or "artemisinin resistan*").ti,ab,kw. (3794)
6 4 and 5 (1070)
7 limit 6 to ((english or french or hindi or italian or spanish) and yr="2014 -Current") (885)

Scopus

(TITLE-ABS-KEY (malaria OR plasmodium) AND TITLE-ABS-KEY (pfkelch* OR pfk13* OR k13 OR kelch* OR "artemisinin resistan*")) AND (LIMIT-TO (PUBYEAR , 2022) OR LIMIT-TO (PUBYEAR , 2021) OR LIMIT-TO (PUBYEAR , 2020) OR LIMIT-TO (PUBYEAR , 2019) OR LIMIT-TO (PUBYEAR , 2018) OR

LIMIT-TO (PUBYEAR , 2017) OR LIMIT-TO (PUBYEAR , 2016) OR LIMIT-TO (PUBYEAR , 2015) OR LIMIT-TO (PUBYEAR , 2014)) AND (LIMIT-TO (LANGUAGE , "English") OR LIMIT-TO (LANGUAGE , "French"))

Web of Science Core Collection
malaria or plasmodium (Topic) AND pfkelch* or pfk13* or k13 or kelch* or "artemisinin resistan*" (Topic)
Refined by Publication Years: 2014 or 2022 or 2021 or 2020 or 2019 or 2018 or 2017 or 2016 or 2015

IndMed
https://www.india.gov.in/gsearch?s=pfkelch&op=Search

Pfkelch, pfk13, k13, kelch – no results
Artemisinin resistance – 1 result

Clinicaltrials.gov

Condition or disease: malaria
Other terms: pfkelch
All Studies

Condition or disease: malaria
Other terms: pfk13
All Studies

Condition or disease: malaria
Other terms: k13
All Studies

Condition or disease: malaria
Other terms: kelch
All Studies

Condition or disease: malaria
Other terms: artemisinin resistance
All Studies

WHO ICTRP https://trialsearch.who.int/
Trials registered from 2014/01/01 - 2022

Condition: Malaria
Intervention: kelch13 or pfk13 or k13 or kelch or artemisinin resistance
ALL studies

Molecular surveillance of Sulfadoxine-Pyrimethamine resistance markers *pfdhps* and *pfdhfr* prevalence in India

Search strategies run on 09/03/2022 by Eli Harriss, a librarian at the Bodleian Health Care Libraries, University of Oxford.

Search Results

Ovid Embase	3646
Ovid Medline	2282
Scopus	4235
Web of Science Core Collection	3513
Clinicaltrials.gov	194
WHO ICTRP	34
Cochrane CENTRAL	369
Total	14278
Total after deduplication	6346

Search Strategies

Database: Embase 1974 to present
Search Strategy:
----
1 exp malaria/ (94183)
2 exp Plasmodium/ (64026)
3 (malaria or plasmodium or falciparum).ti,ab,kw. (116782)
4 1 or 2 or 3 (136184)
5 dihydrofolate reductase/ (7867)
6 dihydropteroate synthase/ (971)
7 ("dihydrofolate reductase" or dhfr).ti,ab,kw. (8512)
8 ("tetrahydrofolate nadp oxidoreductase" or "tetrahydrofolate nicotinamide adenine dinucleotide phosphate oxidoreductase" or "dehydrofolate dehydrogenase" or "dehydrofolate reductase" or "dihydrofolate dehydrogenase" or "dihydrofolic acid reductase" or "dihydrofolic reductase" or "dihydropteroylglutamate reductase" or "e.c. 1.5.1.3" or "e.c. 1.5.1.4" or "folate reductase" or "folic acid reductase" or "folic reductase" or "reductase folate" or "tetrahydrofolate dehydrogenase" or "tetrahydrofolate reductase").ti,ab,kw. (1334)
9 ("dihydropteroate synthase" or dhps).ti,ab,kw. (1698)
10 ("dihydropteroate synthetase" or "dihydropteroate pyrophosphorylase" or "dihydropteroate synthetase" or "e.c. 2.5.1.15").ti,ab,kw. (155)
11 molecular marker/ (15716)
12 (molecular and (resistance or marker* or signature* or surveillance)).ti,ab,kw. (273520)
13 (pfdhps or pfdhfr).ti,ab,kw. (513)
14 pyrimethamine plus sulfadoxine/ (2086)
15 (vivaxine or rimodar or suldox or fansidar or laridox or madomine or methamar or pyralfin or pyrimethamine* or sulphadoxine* or sulfadoxine*).ti,ab,kw. (6463)
16 5 or 6 or 7 or 8 or 9 or 10 or 11 or 12 or 13 or 14 or 15 (294664)
17 4 and 16 (8818)

18 limit 17 to ((english or french or hindi or italian or spanish) and yr="2014 - 2022") (3646)

Database: Medline (Ovid MEDLINE® Epub Ahead of Print, In-Process & Other Non-Indexed Citations, Ovid MEDLINE® Daily and Ovid MEDLINE®) 1946 to present
Search Strategy:
----
1 exp Malaria/ (71077)
2 exp Plasmodium/ (50380)
3 (malaria or plasmodium or falciparum).ti,ab,kw. (103350)
4 1 or 2 or 3 (112384)
5 Tetrahydrofolate Dehydrogenase/ (5916)
6 Dihydropteroate Synthase/ (660)
7 ("dihydrofolate reductase" or dhfr).ti,ab,kw. (7656)
8 ("tetrahydrofolate nadp oxidoreductase" or "tetrahydrofolate nicotinamide adenine dinucleotide phosphate oxidoreductase" or "dehydrofolate dehydrogenase" or "dehydrofolate reductase" or "dihydrofolate dehydrogenase" or "dihydrofolic acid reductase" or "dihydrofolic reductase" or "dihydropteroylglutamate reductase" or "e.c. 1.5.1.3" or "e.c. 1.5.1.4" or "folate reductase" or "folic acid reductase" or "folic reductase" or "reductase folate" or "tetrahydrofolate dehydrogenase" or "tetrahydrofolate reductase").ti,ab,kw. (1145)
9 ("dihydropteroate synthase" or dhps).ti,ab,kw. (1425)
10 ("dihydropteroate synthetase" or "dihydropteroate pyrophosphorylase" or "dihydropteroate synthetase" or "e.c. 2.5.1.15").ti,ab,kw. (134)
11 (molecular and (resistance or marker* or signature* or surveillance)).ti,ab,kw. (200534)
12 (pfdhps or pfdhfr).ti,ab,kw. (380)
13 (vivaxine or rimodar or suldox or fansidar or laridox or madomine or methamar or pyralfin or pyrimethamine* or sulphadoxine* or sulfadoxine*).ti,ab,kw. (5667)
14 5 or 6 or 7 or 8 or 9 or 10 or 11 or 12 or 13 (215432)
15 4 and 14 (6503)
16 limit 15 to (yr="2014 -Current" and (english or french or hindi or italian or spanish)) (2282)

****

Scopus
(TITLE-ABS-KEY (malaria OR plasmodium OR falciparum)) AND ((TITLE-ABS-KEY ("dihydrofolate reductase" OR dhfr OR "tetrahydrofolate nadp oxidoreductase" OR "tetrahydrofolate nicotinamide adenine dinucleotide phosphate oxidoreductase" OR "dehydrofolate dehydrogenase" OR "dehydrofolate reductase" OR "dihydrofolate dehydrogenase" OR "dihydrofolic acid reductase" OR "dihydrofolic reductase" OR "dihydropteroylglutamate reductase" OR "e.c. 1.5.1.3" OR "e.c. 1.5.1.4" OR "folate reductase" OR "folic acid reductase" OR "folic reductase" OR "reductase folate" OR "tetrahydrofolate dehydrogenase" OR "tetrahydrofolate reductase" OR "dihydropteroate synthase" OR dhps OR "dihydropteroate synthetase" OR "dihydropteroate pyrophosphorylase" OR "dihydropteroate synthetase" OR "e.c. 2.5.1.15") OR TITLE-ABS-KEY ((molecular AND (resistance OR marker* OR signature* OR surveillance))) OR TITLE-ABS-KEY (pfdhps OR pfdhfr OR vivaxine OR rimodar OR suldox OR fansidar OR laridox OR madomine OR methamar OR pyralfin OR pyrimethamine* OR sulphadoxine* OR sulfadoxine*))) AND (LIMIT-TO (LANGUAGE , "English") OR LIMIT-TO (LANGUAGE , "French") OR LIMIT-TO (LANGUAGE , "Italian"))

AND (LIMIT-TO (PUBYEAR , 2022) OR LIMIT-TO (PUBYEAR , 2021) OR LIMIT-TO (PUBYEAR , 2020) OR LIMIT-TO (PUBYEAR , 2019) OR LIMIT-TO (PUBYEAR , 2018) OR LIMIT-TO (PUBYEAR , 2017) OR LIMIT-TO (PUBYEAR , 2016) LIMIT-TO (PUBYEAR , 2015) OR LIMIT-TO (PUBYEAR , 2014))

Web of Science Core Collections (All Editions)

#1 malaria OR plasmodium OR falciparum (Topic)
#2 "dihydrofolate reductase" OR dhfr OR "tetrahydrofolate nadp oxidoreductase" OR "tetrahydrofolate nicotinamide adenine dinucleotide phosphate oxidoreductase" OR "dehydrofolate dehydrogenase" OR "dehydrofolate reductase" OR "dihydrofolate dehydrogenase" OR "dihydrofolic acid reductase" OR "dihydrofolic reductase" OR "dihydropteroylglutamate reductase" OR "e.c. 1.5.1.3" OR "e.c. 1.5.1.4" OR "folate reductase" OR "folic acid reductase" OR "folic reductase" OR "reductase folate" OR "tetrahydrofolate dehydrogenase" OR "tetrahydrofolate reductase" OR "dihydropteroate synthase" OR dhps OR "dihydropteroate synthetase" OR "dihydropteroate pyrophosphorylase" OR "dihydropteroate synthetase" OR "e.c. 2.5.1.15" (Topic) or molecular AND (resistance OR marker* OR signature* OR surveillance) (Topic) or pfdhps OR pfdhfr OR vivaxine OR rimodar OR suldox OR fansidar OR laridox OR madomine OR methamar OR pyralfin OR pyrimethamine* OR sulphadoxine* OR sulfadoxine* (Topic)
#3 #1 and #2 and English or French or Spanish (Languages) and 2014 or 2015 or 2016 or 2017 or 2018 or 2019 or 2020 or 2021 or 2022 (Publication Years)

Clinicaltrials.gov

Condition or disease: malaria
Other terms: dihydrofolate reductase
All Studies

Condition or disease: malaria
Other terms: dhfr
All Studies

Condition or disease: malaria
Other terms: dihyropteroate synthase
All Studies

Condition or disease: malaria
Other terms: dhps
All Studies

Condition or disease: malaria
Other terms: Sulfadoxine-pyrimethamine
All Studies

WHO ICTRP https://trialsearch.who.int/
Trials registered from 2014/01/01 - 2022

dihydrofolate reductase

Recruitment status is: ALL

dhfr
Recruitment status is: ALL

dihyropteroate synthase
Recruitment status is: ALL

dhps
Recruitment status is: ALL

Sulfadoxine-pyrimethamine
Recruitment status is: ALL

Cochrane Central Register of Controlled Trials
Issue 2 of 12, February 2022

#1 MeSH descriptor: [Malaria] explode all trees3265
#2 MeSH descriptor: [Plasmodium] explode all trees 987
#3 (malaria or plasmodium or falciparum):ti,ab,kw 7153
#4 #1 or #2 or #3 7153
#5 MeSH descriptor: [Tetrahydrofolate Dehydrogenase] explode all trees 23
#6 MeSH descriptor: [Dihydropteroate Synthase] explode all trees 12
#7 ("dihydrofolate reductase" or dhfr):ti,ab,kw 131
#8 ("tetrahydrofolate nadp oxidoreductase" or "tetrahydrofolate nicotinamide adenine dinucleotide phosphate oxidoreductase" or "dehydrofolate dehydrogenase" or "dehydrofolate reductase" or "dihydrofolate dehydrogenase" or "dihydrofolic acid reductase" or "dihydrofolic reductase" or "dihydropteroylglutamate reductase" or "e.c. 1.5.1.3" or "e.c. 1.5.1.4" or "folate reductase" or "folic acid reductase" or "folic reductase" or "reductase folate" or "tetrahydrofolate dehydrogenase" or "tetrahydrofolate reductase"):ti,ab,kw 45
#9 ("dihydropteroate synthase" or dhps):ti,ab,kw 60
#10 ("dihydropteroate synthetase" or "dihydropteroate pyrophosphorylase" or "dihydropteroate synthetase" or "e.c. 2.5.1.15"):ti,ab,kw 4
#11 (molecular and (resistance or marker* or signature* or surveillance)):ti,ab,kw 4318
#12 (pfdhps or pfdhfr):ti,ab,kw 27
#13 (vivaxine or rimodar or suldox or fansidar or laridox or madomine or methamar or pyralfin or pyrimethamine* or sulphadoxine* or sulfadoxine*):ti,ab,kw 1407
#14 #5 or #6 or #7 or #8 or #9 or #10 or #11 or #12 or #13 5751
#15 #4 and #14 1338
Limited by date: 2014 to 2022

***P. falciparum* resistance markers**	**Study inclusion criteria**	**Study exclusion criteria**
pfk13	• Indian Studies involving humans of all age groups and gender<br>• *pfk13* genotypes assessed by sequencing at least one *P. falciparum* isolate/infection<br>• Original data<br>• Baseline/pre-treatment infections<br>• Marker prevalence must be linked to the geographical site	• Non-human *P. falciparum* infections<br>• Publication not accessible<br>• Publications in other languages than English, French, Spain, Italian, or Hindi<br>• Non-primary research studies/Review<br>• Cultured strains, genetically manipulated strains, strains that have been adapted to long-term culture in vitro (not representative of original infection)<br>• Parasites isolated from other tissues than blood (for example placenta)<br>• Post-treatment infections<br>• Biased selection of baseline samples (e.g. only treatment failures, half resistant and half sensitive in vitro tested isolates)<br>• Regionally pooled marker prevalence data, where the origin of infection cannot be linked to a geographical site.<br>• Marker prevalence (%) presented in the article without sample size.<br>• Full text could not be found
pfdhps/pfdhfr	• Indian studies involving humans of all age groups and gender<br>• At least one *pfdhps* or *pfdhfr* genotype/haplotype/copy number from isolate/infection<br>• Original data<br>• Baseline/pre-treatment infections<br>• Marker prevalence must be linked to the study site/country	• Non-human *P. falciparum* infections<br>• Publication not accessible<br>• Publications in other languages than English, French, Spain, Italian, or Hindi<br>• Non-primary research studies and Review articles<br>• Cultured strains, genetically manipulated strains, strains that have been adapted to long-term culture in vitro (not representative of original infection)<br>• Parasites isolated from other tissues than blood (for example placenta)<br>• Post-treatment infections<br>• Biased selection of baseline samples (e.g. only treatment failures, half resistant and half sensitive in vitro tested isolates)<br>• Regionally pooled marker prevalence data, where the origin of infection cannot be deduced on at least the country level.<br>• Marker prevalence (%) presented in the article without sample size.

Supplementary Table 1: Inclusion and exclusion criteria for selection of *pfk13* and *pfdhps/pfdhfr* resistance marker prevalence studies for the systematic review.

***P. falciparum* resistance markers**	**PubMed Id**	**Studies excluded at the full-text screening stage**	**Brief reasons**
pfk13	NA	1. Molecular evidence for *Plasmodium falciparum* resistance to sulfadoxine-pyrimethamine but the absence of k-13 mutations in Mangalore, southern India	Missing information
	NA	2. Slow parasite clearance, absent k-13 propeller gene polymorphisms and adequate artesunate levels among patients with severe malaria: a prospective observational cohort study from south India	Missing information on the number of sequenced samples
	NA	3. High-throughput genomic surveillance Plasmodium infections in India	Missing information
Pfdhps/pfdhfr	26068343	4. Population genetic study of Plasmodium falciparum parasites pertaining to *pfdhps* gene sequence in malaria-endemic areas of Assam	Marker prevalence data is not available
	NA	5. Analysis of sulphadoxine-pyrimethamine drug resistance in Plasmodium falciparum from Khurda District of Odisha, India	RFLP, not a sequencing method in inclusion criteria
	30428283	6. Evidence of artemisinin-resistant Plasmodium falciparum malaria in Eastern India	Does not contain *pfdhps* or *pfdhfr* genotypes
	27448953	7. Polymorphisms in pfcrt and pfmdr-1 genes after five years of withdrawal of chloroquine for the treatment of Plasmodium falciparum malaria in West Bengal, India	Does not contain *pfdhps* or *pfdhfr* genotypes
	31548652	8. Characterization of drug resistance and genetic diversity of Plasmodium falciparum parasites from Tripura, Northeast India	Does not contain *pfdhps* or *pfdhfr* genotypes
	28303837	9. Prevalence of multiple drug-resistant Plasmodium falciparum malaria cases in Northeast India	missing information; did not understand the presentation of the results
	32280801	10. Comparative analysis of Plasmodium falciparum dihydrofolate-reductase gene sequences from different regions of India	Bioinformatics analysis of previously published genotypes
	NA	11. High-throughput genomic surveillance of plasmodium infections in India	Missing information
	27485211	12. Low prevalence of dihydrofolate	Only *P. vivax* data, no *P.*

S. No.	References	No. of samples	Sequencing method
1	Bharti et al.[1]	186	Sanger sequencing
2	Mishra et al.[2]	384	Sanger sequencing
3	Rao et al.[3]	16	Ion Torrent
4	Ozarkar et al.[4]	63	Nanodrop 1000
5	Das et al.[5]	180	Sanger sequencing
6	Mishra et al.[6]	135	Sanger sequencing
7	Chakrabarti et al.[7]	22	Not mentioned
8	Saksena et al.[8]	1	Sanger sequencing
9	Das et al.[9]	136	Not mentioned
10	Wedam et al.[10]	112	Sanger sequencing
11	Rana et al.[11]	178	Sanger sequencing
12	Pathak et al.[12]	179	Not mentioned
13	Prosser et al.[13]	2	Sanger sequencing
14	Krishna et al.[14]	308	Sanger sequencing
15	Kumar et al.[15]	122	Sanger sequencing
16	Chatterjee et al.[16]	51	Not mentioned
17	Mishra et al.[17]	254	De novo NGS
18	Ashley et al.[18]	1	Illumina sequencing
19	Das et al.[19]	226	Sanger sequencing
20	Miraclin et al.[20]	42	Sanger sequencing
21	Patgiri et al.[21]	71	Sanger sequencing
22	Unpublished study	371	Not mentioned

Supplementary Table 3: *pfk13* prevalence studies with sample size, and type of sequencing method used. nsSNP: Nonsynonymous Single Nucleotide Polymorphism.

References:

1. Bharti PK, Shukla MM, Ringwald P, Krishna S, Singh PP, Yadav A, Mishra S, Gahlot U, Malaiya JP, Kumar A, Prasad S, Baghel P, Singh M, Vadadi J, Singh MP, et al., 2016. Therapeutic efficacy of artemether–lumefantrine for the treatment of uncomplicated Plasmodium falciparum malaria from three highly malarious states in India. *Malar J 15*: 498
2. Mishra N, Prajapati SK, Kaitholia K, Bharti RS, Srivastava B, Phookan S, Anvikar AR, Dev V, Sonal GS, Dhariwal AC, White NJ, Valecha N., 2015. Surveillance of Artemisinin Resistance in Plasmodium falciparum in India Using the kelch13 Molecular Marker. *Antimicrob Agents Chemother 59*: 2548–2553
3. Rao PN, Uplekar S, Kayal S, Mallick PK, Bandyopadhyay N, Kale S, Singh OP, Mohanty A, Mohanty S, Wassmer SC, Carlton JM., 2016. A Method for Amplicon Deep Sequencing of Drug Resistance Genes in Plasmodium falciparum Clinical Isolates from India. *J Clin Microbiol 54*: 1500–1511
4. Ozarkar A, Kanyal A, Dass S, Deshpande P, Deobagkar D, Karmodiya K., 2021. Analysis of drug resistance marker genes of Plasmodium falciparum after implementation of artemisinin-based combination therapy in Pune district, India. *J Biosci 46*: 77

5. Das S, Kar A, Manna S, Mandal S, Mandal S, Das S, Saha B, Hati AK., 2021. Artemisinin combination therapy fails even in the absence of Plasmodium falciparum kelch13 gene polymorphism in Central India. *Sci Rep 11*: 9946
6. Mishra S, Bharti PK, Shukla MM, Ali NA, Kashyotia SS, Kumar A, Dhariwal AC, Singh N., 2017. Clinical and molecular monitoring of *Plasmodium falciparum* resistance to antimalarial drug (artesunate+sulphadoxine-pyrimethamine) in two highly malarious district of Madhya Pradesh, Central India from 2012–2014. *Pathogens and Global Health 111*: 186–194
7. Chakrabarti R, White J, Babar PH, Kumar S, Mudeppa DG, Mascarenhas A, Pereira L, Dash R, Maki JN, Sharma A, Gogoi K, Sarma DK, Pal Bhowmick I, Manoharan SK, Gomes E, et al., 2019. Decreased *In Vitro* Artemisinin Sensitivity of *Plasmodium falciparum* across India. *Antimicrob Agents Chemother 63*: e00101-19
8. Saksena R, Matlani M, Singh V, Kumar A, Anveshi A, Kumar D, Gaind R., 2017. Early treatment failure in concurrent dengue and mixed malaria species infection with suspected resistance to artemisinin combination therapy from a tertiary care center in Delhi: a case report. *Int Med Case Rep J 10*: 289–294
9. Das S, Saha B, Hati AK, Roy S., 2018. Evidence of Artemisinin-Resistant Plasmodium falciparum Malaria in Eastern India. *New England Journal of Medicine 379*: 1962–1964
10. Wedam J, Tacoli C, Gai PP, Siegert K, Kulkarni SS, Rasalkar R, Boloor A, Jain A, Mahabala C, Baliga S, Shenoy D, Devi R, Gai P, Mockenhaupt FP., 2018. Molecular Evidence for Plasmodium falciparum Resistance to Sulfadoxine–Pyrimethamine but Absence of K13 Mutations in Mangaluru, Southwestern India. *The American Journal of Tropical Medicine and Hygiene 99*: 1508–1510
11. Rana R, Ranjit M, Bal M, Khuntia HK, Pati S, Krishna S, Das A., 2020. Sequence Analysis of the *K13* -Propeller Gene in Artemisinin Challenging *Plasmodium falciparum* Isolates from Malaria Endemic Areas of Odisha, India: A Molecular Surveillance Study. *BioMed Research International 2020*: 1–6
12. Pathak A, Mårtensson A, Gawariker S, Sharma A, Diwan V, Purohit M, Ursing J., 2020. Stable high frequencies of sulfadoxine–pyrimethamine resistance associated mutations and absence of K13 mutations in Plasmodium falciparum 3 and 4 years after the introduction of artesunate plus sulfadoxine–pyrimethamine in Ujjain, Madhya Pradesh, India. *Malar J 19*: 290
13. Prosser C, Meyer W, Ellis J, Lee R., 2018. Resistance screening and trend analysis of imported falciparum malaria in NSW, Australia (2010 to 2016). *PLoS ONE 13*: e0197369
14. Krishna S, Mishra S, Tiwari P, Vishwakarma AK, Khandai S, Shrivastava S, Verma AK, Tiwari S, Barman H, Jhariya S, Tiwari P, Tidgam AS, Varun BM, Singh S, Yerane N, et al., 2021. Therapeutic efficacy of artemether-lumefantrine for the treatment of uncomplicated Plasmodium falciparum malaria in four malaria endemic states of India. *Malar J 20*: 229
15. Kumar A, Singh SP, Bhatt R, Singh V., 2019. Genetic profiling of the Plasmodium falciparum parasite population in uncomplicated malaria from India. *Malar J 18*: 385
16. Chatterjee M, Ganguly S, Saha P, Bankura B, Basu N, Das M, Guha SK, Maji AK., 2015. No Polymorphism in *Plasmodium falciparum K13* Propeller Gene in Clinical Isolates from Kolkata, India. *Journal of Pathogens 2015*: 1–4
17. Mishra N, Bharti RS, Mallick P, Singh OP, Srivastava B, Rana R, Phookan S, Gupta HP, Ringwald P, Valecha N., 2016. Emerging polymorphisms in falciparum Kelch 13 gene in Northeastern region of India. *Malar J 15*: 583

18. Ashley EA, Dhorda M, Fairhurst RM, Amaratunga C, Lim P, Suon S, Sreng S, Anderson JM, Mao S, Sam B, Sopha C, Chuor CM, Nguon C, Sovannaroth S, Pukrittayakamee S, et al., 2014. Spread of Artemisinin Resistance in *Plasmodium falciparum* Malaria. *N Engl J Med 371*: 411–423
19. Das S, Manna S, Saha B, Hati AK, Roy S., 2019. Novel pfkelch13 Gene Polymorphism Associates With Artemisinin Resistance in Eastern India. *Clinical Infectious Diseases 69*: 1144–1152
20. Miraclin TA, Mathew BS, Mammen JJ, Ramachandran SV, Kumar S, Bhattacharjee S, Sudarsanam TD, Sathyendra S, Prabhakar Abhilash KP, Jayaseelan V, Rupali P., 2019. Slow parasite clearance, absent K13-propeller gene polymorphisms and adequate artesunate levels among patients with malaria: A pilot study from southern India. *Natl Med J India 32*: 200–206
21. Patgiri SJ, Sarma K, Sarmah N, Bhattacharyya N, Sarma DK, Nirmolia T, Bhattacharyya DR, Mohapatra PK, Bansal D, Bharti PK, Sehgal R, Mahanta J, Sultan AA., 2019. Characterization of drug resistance and genetic diversity of Plasmodium falciparum parasites from Tripura, Northeast India. *Sci Rep 9*: 13704

S. No.	References	No. of samples (*pfdhps/pfdhfr*)	Sequencing method
1	Kar et al.[1]	229	de novo NGS
2	Sharma et al.[2]	46	Sanger sequencing
3	Patel et al.[3]	163	Sanger sequencing
4	Mishra et al.[4]	155	Not mentioned
5	Sarmah et al.[5]	75	Sanger sequencing
6	Das et al.[6]	176	Sanger sequencing
7	Kumar et al.[7]	185	Sanger sequencing
8	Das et al.[8]	226	Sanger sequencing
9	Das Sutar et al.[9]	201	Not mentioned
10	Das et al.[10]	90	Enzymatic digestion
11	Mishra et al.[11]	*pfdhps*:327,*pfdhfr*:292	Not mentioned
12	Das et al.[12]	180	Sanger sequencing
13	Sharma et al.[13]	55	Sanger sequencing
14	Sharma et al.[14]	58	Not mentioned
15	Pathak et al.[15]	*pfdhps*:105,*pfdhfr*:104	Not mentioned
16	Pathak et al.[16]	*pfdhps*:77,*pfdhfr*:78	de novo NGS
17	Wedam et al.[17]	107	Not mentioned
18	Wedam et al[18]	112	Sanger sequencing
19	Sharma et al.[19]	94	de novo NGS
20	Mishra et al.[20]	*pfdhps*:216,*pfdhfr*:232	Sanger sequencing
21	Mohapatra et al.[21]	*pfdhps*:173,*pfdhfr*:168	PCR-RFLP
22	Rao et al.[22]	16	Ion torrent
23	Ozarkar et al.[23]	63	Nanodrop 1000
24	Chatterjee et al.[24]	113	Not mentioned
25	Tyagi et al.[25]	*pfdhfr*:39	Sanger sequencing
26	Srivastava et al.[26]	*pfdhps*:88,*pfdhfr*:136	Mutation specific PCR assays
27	Das et al.[27]	*pfdhps*:127	Sanger sequencing
28	Sharma et al.[28]	217	Sanger sequencing
29	Ganguly et al.[29]	*pfdhps*:26,*pfdhfr*:24	Sanger sequencing
30	Kumar et al.[30]	*pfdhps*:121,*pfdhfr*:122	Sanger sequencing
31	Unpublished	*pfdhps*:401,*pfdhfr*:392	Not mentioned

Supplementray Table 4: *pfdhps/pfdhfr* prevalence studies with sample size, and type of sequencing method used. nsSNP: Non synonymous Single Nucleotide Polymorphism.

References:

1. Kar NP, Chauhan K, Nanda N, Kumar A, Carlton JM, Das A., 2016. Comparative assessment on the prevalence of mutations in the Plasmodium falciparum drug-resistant genes in two different ecotypes of Odisha state, India. *Infection, Genetics and Evolution 41*: 47–55
2. Sharma J, Khan SA, Dutta P, Mahanta J., 2015. Molecular determination of antifolate resistance associated point mutations in Plasmodium falciparum dihydrofolate reductase (dhfr) and

dihydropteroate synthetase (dhps) genes among the field samples in Arunachal Pradesh. *J Vector Borne Dis 52*: 116–121
3. Patel P, Bharti PK, Bansal D, Ali NA, Raman RK, Mohapatra PK, Sehgal R, Mahanta J, Sultan AA, Singh N., 2017. Prevalence of mutations linked to antimalarial resistance in Plasmodium falciparum from Chhattisgarh, Central India: A malaria elimination point of view. *Sci Rep 7*: 16690
4. Mishra N, Kaitholia K, Srivastava B, Shah NK, Narayan JP, Dev V, Phookan S, Anvikar AR, Rana R, Bharti RS, Sonal GS, Dhariwal AC, Valecha N., 2014. Declining efficacy of artesunate plus sulphadoxine-pyrimethamine in northeastern India. *Malar J 13*: 284
5. Sarmah NP, Sarma K, Bhattacharyya DR, Sultan AA, Bansal D, Singh N, Bharti PK, Sehgal R, Mohapatra PK, Parida P, Mahanta J., 2017. Antifolate drug resistance: Novel mutations and haplotype distribution in dhps and dhfr from Northeast India. *J Biosci 42*: 531–535
6. Das S, Chakraborty SP, Hati A, Roy S., 2013. Malaria treatment failure with novel mutation in the Plasmodium falciparum dihydrofolate reductase (pfdhfr) gene in Kolkata, West Bengal, India. *International Journal of Antimicrobial Agents 41*: 447–451
7. Kumar A, Moirangthem R, Gahlawat SK, Chandra J, Gupta P, Valecha N, Anvikar A, Singh V., 2015. Emergence of sulfadoxine–pyrimethamine resistance in Indian isolates of Plasmodium falciparum in the last two decades. *Infection, Genetics and Evolution 36*: 190–198
8. Das S, Manna S, Saha B, Hati AK, Roy S., 2019. Novel pfkelch13 Gene Polymorphism Associates With Artemisinin Resistance in Eastern India. *Clinical Infectious Diseases 69*: 1144–1152
9. Das Sutar SK, Dhangadamajhi G, Kar SK, Ranjit M., 2013. Molecular monitoring of antimalarial drug resistance among Plasmodium falciparum field isolates from Odisha, India. *Acta Tropica 126*: 84–87
10. Das S, Chakraborty SP, Tripathy S, Hati A, Roy S., 2012. Novel quadruple mutations in dihydropteroate synthase genes of *Plasmodium falciparum* in West Bengal, India: **Quadruple mutations of *pfdhps* gene in malaria** . *Tropical Medicine & International Health 17*: 1329–1334
11. Mishra N, Srivastava B, Bharti RS, Rana R, Kaitholia K, Anvikar AR, Das MK, Ghosh SK, Bhatt RM, Tyagi PK, Dev V, Phookan S, Wattal SL, Sonal GS, Dhariwal AC, et al., 2016. Monitoring the efficacy of antimalarial medicines in India via sentinel sites: Outcomes and risk factors for treatment failure. *J Vector Borne Dis 53*: 168–178
12. Das S, Kar A, Manna S, Mandal S, Mandal S, Das S, Saha B, Hati AK., 2021. Artemisinin combination therapy fails even in the absence of Plasmodium falciparum kelch13 gene polymorphism in Central India. *Sci Rep 11*: 9946
13. Sharma D, Lather M, Dykes CL, Dang AS, Adak T, Singh OP., 2016. Disagreement in genotyping results of drug resistance alleles of the Plasmodium falciparum dihydrofolate reductase (Pfdhfr) gene by allele-specific PCR (ASPCR) assays and Sanger sequencing. *Parasitol Res 115*: 323–328
14. Sharma J, Dutta P, Khan SA., 2016. Antifolate resistance associated point mutations among asymptomatic malaria cases. *J Vector Borne Dis 53*: 81–83
15. Pathak A, Mårtensson A, Gawariker S, Sharma A, Diwan V, Purohit M, Ursing J., 2020. Stable high frequencies of sulfadoxine–pyrimethamine resistance associated mutations and absence of K13 mutations in Plasmodium falciparum 3 and 4 years after the introduction of artesunate plus sulfadoxine–pyrimethamine in Ujjain, Madhya Pradesh, India. *Malar J 19*: 290
16. Pathak A, Mårtensson A, Gawariker S, Mandliya J, Sharma A, Diwan V, Ursing J., 2014. Characterization of drug resistance associated genetic polymorphisms among Plasmodium falciparum field isolates in Ujjain, Madhya Pradesh, India. *Malar J 13*: 182
17. Jakob Wedam1, Costanza Tacoli1, Prabhanjan P. Gai1, Konrad Siegert1, Suyamindra Kulkarni2, Rashmi Rasalkar2, Animesh Jain3, Chakrapani Mahabala3, Shantaram Baliga3, Damodara

Shenoy3, Pramod Gai2, Rajeshwari Devi4, Frank P. Mockenhaupt1. MOLECULAR EVIDENCE FOR PLASMODIUM FALCIPARUM RESISTANCE TO SULFADOXINE-PYRIMETHAMINE BUT ABSENCE OF K13 MUTATIONS IN MANGALORE, SOUTHERN INDIA. *AJTMH*

18. Wedam J, Tacoli C, Gai PP, Siegert K, Kulkarni SS, Rasalkar R, Boloor A, Jain A, Mahabala C, Baliga S, Shenoy D, Devi R, Gai P, Mockenhaupt FP., 2018. Molecular Evidence for Plasmodium falciparum Resistance to Sulfadoxine–Pyrimethamine but Absence of K13 Mutations in Mangaluru, Southwestern India. *The American Journal of Tropical Medicine and Hygiene 99*: 1508–1510
19. Dutta P, Khan S, Soni M, Dey D, Mahanta J, Sharma J., 2015. Genetic polymorphisms associated with sulphadoxine-pyrimethamine drug resistance among Plasmodium falciparum field isolates in malaria endemic areas of Assam. *J Postgrad Med 61*: 9
20. Mishra S, Bharti PK, Shukla MM, Ali NA, Kashyotia SS, Kumar A, Dhariwal AC, Singh N., 2017. Clinical and molecular monitoring of *Plasmodium falciparum* resistance to antimalarial drug (artesunate+sulphadoxine-pyrimethamine) in two highly malarious district of Madhya Pradesh, Central India from 2012–2014. *Pathogens and Global Health 111*: 186–194
21. Mohapatra PK, Sarma DK, Prakash A, Bora K, Ahmed MdA, Sarma B, Goswami BK, Bhattacharyya DR, Mahanta J., 2014. Molecular Evidence of Increased Resistance to Anti-Folate Drugs in Plasmodium falciparum in North-East India: A Signal for Potential Failure of Artemisinin Plus Sulphadoxine-Pyrimethamine Combination Therapy. *PLoS ONE 9*: e105562
22. Rao PN, Uplekar S, Kayal S, Mallick PK, Bandyopadhyay N, Kale S, Singh OP, Mohanty A, Mohanty S, Wassmer SC, Carlton JM., 2016. A Method for Amplicon Deep Sequencing of Drug Resistance Genes in Plasmodium falciparum Clinical Isolates from India. *J Clin Microbiol 54*: 1500–1511
23. Ozarkar A, Kanyal A, Dass S, Deshpande P, Deobagkar D, Karmodiya K., 2021. Analysis of drug resistance marker genes of Plasmodium falciparum after implementation of artemisinin-based combination therapy in Pune district, India. *J Biosci 46*: 77
24. Chatterjee M, Ganguly S, Saha P, Guha SK, Maji AK., 2017. Polymorphisms in pfdhfr and pfdhps genes after five years of artemisinin combination therapy (ACT) implementation from urban Kolkata, India. *Infection, Genetics and Evolution 53*: 155–159
25. Tyagi RK, Das MK, Singh SS, Sharma YD., 2013. Discordance in drug resistance-associated mutation patterns in marker genes of Plasmodium falciparum and Plasmodium knowlesi during coinfections. *Journal of Antimicrobial Chemotherapy 68*: 1081–1088
26. Srivastava P, Ratha J, Shah NK, Mishra N, Anvikar AR, Sharma SK, Das MK, Srivastava B, Valecha N., 2013. A clinical and molecular study of artesunate + sulphadoxine-pyrimethamine in three districts of central and eastern India. *Malar J 12*: 247
27. Das MK, Chetry S, Kalita MC, Dutta P., 2016. Evidence of triple mutant Pf dhps IS GNG A haplotype in Plasmodium falciparum isolates from North-east India: An analysis of sulfadoxine resistant haplotype selection. *Genomics Data 10*: 144–150
28. Sharma D, Lather M, Mallick PK, Adak T, Dang AS, Valecha N, Singh OP., 2015. Polymorphism in drug resistance genes dihydrofolate reductase and dihydropteroate synthase in Plasmodium falciparum in some states of India. *Parasit Vectors 8*: 471
29. Ganguly S, Saha P, Guha SK, Biswas A, Das S, Kundu PK, Maji AK., 2013. High Prevalence of Asymptomatic Malaria in a Tribal Population in Eastern India. *J Clin Microbiol 51*: 1439–1444
30. Kumar A, Singh SP, Bhatt R, Singh V., 2019. Genetic profiling of the Plasmodium falciparum parasite population in uncomplicated malaria from India. *Malar J 18*: 385

S. No.	References	No. of samples (*pfdhps*/*pfdhfr*)	Sequencing method
1	Kar et al.[16]	229	de novo NGS
2	Sharma et al.[10]	46	Sanger sequencing
3	Patel et al.[17]	163	Sanger sequencing
4	Mishra et al.[11]	155	Not mentioned
5	Sarmah et al.[58]	75	Sanger sequencing
6	Das et al.[59]	176	Sanger sequencing
7	Kumar et al.[60]	185	Sanger sequencing
8	Das et al.[39]	226	Sanger sequencing
9	Das Sutar et al.[18]	201	Not mentioned
10	Das et al.[19]	90	Enzymatic digestion
11	Mishra et al.[12]	*pfdhps*:327,*pfdhfr*:292	Not mentioned
12	Das et al.[15]	180	Sanger sequencing
13	Sharma et al.[61]	55	Sanger sequencing
14	Sharma et al.[62]	58	Not mentioned
15	Pathak et al.[20]	*pfdhps*:105,*pfdhfr*:104	Not mentioned
16	Pathak et al.[21]	*pfdhps*:77,*pfdhfr*:78	de novo NGS
17	Wedam et al.[63]	107	Not mentioned
18	Wedam et al[22]	112	Sanger sequencing
19	Sharma et al.[64]	94	de novo NGS
20	Mishra et al.[43]	*pfdhps*:216,*pfdhfr*:232	Sanger sequencing
21	Mohapatra et al.[23]	*pfdhps*:173,*pfdhfr*:168	PCR-RFLP
22	Rao et al.[50]	16	Ion torrent
23	Ozarkar et al.[35]	63	Nanodrop 1000
24	Chatterjee et al.[65]	113	Not mentioned
25	Tyagi et al.[66]	*pfdhfr*:39	Sanger sequencing
26	Srivastava et al.[45]	*pfdhps*:88,*pfdhfr*:136	Mutation specific PCR assays
27	Das et al.[67]	*pfdhps*:127	Sanger sequencing
28	Sharma et al.[37]	217	Sanger sequencing
29	Ganguly et al.[46]	*pfdhps*:26,*pfdhfr*:24	Sanger sequencing
30	Kumar et al.[54]	*pfdhps*:121,*pfdhfr*:122	Sanger sequencing
31	Unpublished	*pfdhps*:401,*pfdhfr*:392	Not mentioned

Supplementray Table 5: *pfdhps/pfdhfr* prevalence studies with sample size, and type of sequencing method used. nsSNP: Non synonymous Single Nucleotide Polymorphism.
